# Gene expression of calcium transporters Calbindin-D28K and TRPV6 in Japanese quails (*Coturnix japonica*) subjected to phytase super-dosing and under different temperatures.

**DOI:** 10.1016/j.psj.2025.104937

**Published:** 2025-02-21

**Authors:** Apolônio Gomes Ribeiro, Raiane dos Santos Silva, Clara Virgínia Batista de Vasconcelos Alves, Danila Barreiro Campos, Dayane Albuquerque da Silva, Júlio Cézar dos Santos Nascimento, Edijanio Galdino da Silva, Edilson Paes Saraiva, Fernando Guilherme Perazzo Costa, Walter Esfrain Pereira, Lucas Rannier Ribeiro Antonino Carvalho, Ricardo Romão Guerra

**Affiliations:** a Universidade Federal da Paraíba, Animal Science Department, Areia-PB, Brazil; b Universidade Federal da Paraíba, Departament of Veterinary Sciences, Areia-PB, Brazil; c Universidade Federal Rural de Pernambuco, Animal Science Department, Recife-PE, Brazil; d Universidade Federal da Paraíba, Department of Fundamental and Social Sciences, Areia-PB, Brazil; e Department of Physiology and Pharmacology Karolinska Institutet - Stockholm Sweden Biomedicum, 5B, Solnavägen 9, S-171 77, Stockholm, Sweden

**Keywords:** Calbindin-D28 K, Calcium transporters, Heat stress, Phytase supplementation, TRPV6

## Abstract

This study aimed to evaluate the effects of phytase super-dosing in the diet of laying quails subjected to different temperatures, their performance, blood biochemistry, and gene expression of the epithelial calcium transporters Calbindin-D28 K and TRPV6. Seven hundred and twenty (720) Japanese quails in the production phase were distributed in a completely randomized design, in a 5 × 3 factorial scheme, with five levels of phytase supplementation (0, 500, 1000, 1500 and 3000 FTU/kg) and three temperatures (24°C, 30°C and 36°C), totaling 15 treatments, and six replicates of eight birds each. The study began at 8 weeks of age and continued for two 21-day cycles, totaling 42 days of experiment. Performance parameters, serum biochemistry, and gene expression of calbindin-D28 K and TRPV6 were measured through real-time PCR. The data were subjected to analysis of variance, Tukey, and regression. Birds kept at 36°C showed lower feed intake than those kept at 24°C and 30°C. At 30°C, birds had lower serum uric acid levels than birds at 36°C, and higher total egg production, egg mass, and Calbindin-D28 K gene expression in the uterus than those at 24°C and 36°C. Birds kept at 24°C had higher serum calcium and phosphorus levels than those kept at 30°C and 36°C. At a supplementation level of 1500 FTU, phytase provided greater eggshell thickness in quails kept at 36°C and greater calbindin-D28 K gene expression in the kidney. Therefore, the use of 1500 FTU of phytase is recommended for Japanese quail exposed to high temperatures, since phytase overdosage has been shown to be effective in mitigating the negative effects of heat stress.

## Introduction

In tropical climates, widely present in much of Brazil, laying hens face significant challenges that weaken their performance (Silva et al., 2022). High temperatures have had an adverse impact on the zootechnical indices of birds, resulting not only in an increase in the mortality rate, but also negatively affecting the productive and economic results in the poultry industry (Franzini et al., 2022). This condition is especially evident in the breeding of laying quails (*Coturnix japonica*), where eggshell quality is impaired due to calcium restriction, which is negatively impacted by increased temperature (Ebeid et al., 2012; Moraes et al., 2021).

Calcium is predominantly acquired through intestinal absorption, and through bone and renal reabsorption (Beggs and Alexander, 2017). These processes involve the efficient transfer of calcium from the blood to the uterus, with the assistance of two essential calcium transporters in the epithelium, called transient receptor potential vanilloid channel type 6 (TRPV6) and Calbindin-D28k. These transporters play a crucial role in facilitating the mobilization of calcium through intestinal cells (Moraes et al., 2021).

When birds are exposed to high temperature heat stress, they suffer physiological and behavioral changes. These changes have a pronounced impact on food intake and result in structural changes in the intestinal epithelium, impairing the digestibility and absorption of nutrients (Rostagno, 2020). In addition, it is important to consider the adverse effects of anti-nutritional factors such as phytate. This compound not only makes phosphorus and calcium unavailable, but also negatively influences the digestion and absorption of other minerals and amino acids, as well as affecting the energy efficiency of diets (Babatunde et al., 2019; Bernardes et al., 2022; Dallmann et al., 2023). Heat stress, combined with the antinutrional factors of phytate, exacerbate the challenges faced by tropical poultry production, and affect the health and performance of laying hens.

Phytase (*myo-inositol hexaphosphate phosphohydrolase - IP6*) belongs to a class of exogenous enzymes whose function is to degrade the phytate molecule (*inositol hexa-phosphate, IP6*) present in diet ingredients, releasing phosphorus, calcium, and other nutrients to be used by animals (Hirvonen et al., 2019; Sena et al., 2020). The use of higher than usual amounts of phytase is called super-dosing, and has attracted the attention of researchers, since its administration allows for faster and more effective degradation of the phytate molecule present in diets, resulting in the subsequent release of minerals and other nutrients. (Farias et al., 2021).

Studies have demonstrated the beneficial effects of using phytase super-dosing on animal performance (Pirgozliev et al., 2011; Pieniazek et al., 2017; Farias et al., 2021; Martínez-Vallespín et al., 2022), on egg quality, especially shells (Rojas et al., 2017; Farias et al., 2021), and on bone characteristics (Manobhavan et al., 2016; Sharma et al., 2016). It is worth mentioning that these studies were carried out with broilers and laying hens. It is essential to conduct studies evaluating the effects of an super-dosing of phytase in laying quails subjected to high temperatures. In this regard, the objective of this study was to evaluate the effects of an super-dosing of phytase on the parameters of productive performance, serum biochemistry, and gene expression of the calcium transporters Calbindin-D28k and TRPV6 in laying quails subjected to thermoneutral conditions and heat stress.

## Material and methods

### Experimental site and ethics committee

The experiment was conducted in the climatic chambers belonging to the Department of Animal Science at the Federal University of Paraíba. The project had ethical approval from the Animal Care and Use Committee (CEUA) of the Federal University of Paraíba, Brazil, under the protocol process number (3695120121). Euthanasia was performed by electronarcosis in accordance with the institution's CEUA guidelines.

### Animals and experimental design

Seven hundred and twenty (720) Japanese quails in the production phase were used, starting at the eighth week of life, with the egg yield percentage above 95 %. They were distributed in a completely randomized design in a 5 × 3 factorial scheme, with five phytase levels (0, 500, 1000, 1500, and 3000 FTU) and three temperature ranges (24, 30, and 36°C), with six replicates of eight birds each (48 animals per treatment), representing thermoneutral (24°C) and thermal stress (30 and 36°C) ranges, totaling fifteen treatments. The quails began the treatments at eight weeks of age and remained for two cycles of 21 days, totaling 42 days of experiment.

### Housing

The birds were housed in 3 climatic 19.71 m² chambers. Each chamber was equipped with 30 metal cages (55 × 35 × 27 cm), with 1 nipple drinker and 1 trough feeder per cage. The chambers had an air conditioning system, heater, humidifier, dehumidifier, exhaust fan, thermostats, and lighting system. The size of birds/cage was 481.25 cm2/bird. The lighting program provided was 17 h of (artificial) light/day throughout the experimental period.

### Diets and experimental treatment

The diets were formulated according to the recommendations of the Brazilian Tables for Poultry and Swine (Rostagno et al., 2017), only varying with phytase supplementation. All diets were formulated with reduced calcium and phosphorus concentrations according to provisions of the enzyme matrix for dietary phytase at 500FTU (0.165 % Ca - 0.150 % P) (Table 1).

**Table 1:** Experimental diets containing five levels of Phytase (0, 500, 1000, 1500, and 3000 FTU) and reduction in phosphorus and calcium levels taking into account the 500 FTU matrix of the enzyme, for Japanese quails in the laying phase.

Treatments		T1	T2	T3	T4	T5
Ingredientes	Unit	0FTU	500FTU	1000FTU	1500FTU	3000FTU
Corn - 7,88 %	g/kg	597	597	597	597	597
Soybean meal 45,22 %	g/kg	305	305	305	305	305
Soybean oil	g/kg	6.67	6.67	6.67	6.67	6.67
DL-methionine	g/kg	3.98	3.98	3.98	3.98	3.98
L-Lysine HCl	g/kg	2.65	2.65	2.65	2.65	2.65
L-threonine	g/kg	0.35	0.35	0.35	0.35	0.35
Limestone	g/kg	74.37	74.37	74.37	74.37	74.37
Dicalcium Phosphate 18,5 %	g/kg	4.00	4.00	4.00	4.00	4.00
Common Salt	g/kg	3.45	3.45	3.45	3.45	3.45
Mineral premix 1	g/kg	0.50	0.50	0.50	0.50	0.50
Vitamin premix 2	g/kg	0.25	0.25	0.25	0.25	0.25
Choline	g/kg	0.70	0.70	0.70	0.70	0.70
Antioxidante	g/kg	0.10	0.10	0.10	0.10	0.10
Inert 3	g/kg	0.60	0.50	0.40	0.30	0.00
Phytase 4	g/kg	0.00	0.10	0.20	0.30	0.60
Total		1000	1000	1000	1000	1000
**Nutrients**	**Unit**					
Phytase	FTU/kg	0	500	1000	1500	3000
Metabolizable energy	kcal/kg	2800	2800	2800	2800	2800
Crude protein	g/kg	190.00	190.00	190.00	190.00	190.00
Calcium	g/kg	29.93	29.93	29.93	29.93	29.93
Phosphorus total	g/kg	3.94	3.94	3.94	3.94	3.94
Available phosphorus	g/kg	1.77	1.77	1.77	1.77	1.77
Potassium	g/kg	7.32	7.32	7.32	7.32	7.32
Sodium	g/kg	1.55	1.55	1.55	1.55	1.55
Chlorine	g/kg	3.19	3.19	3.19	3.19	3.19
Mogin number	mEq/kg	164.59	164.59	164.59	164.59	164.59
Digestible amino acid (%)						
Digestible Methionine	g/kg	6.47	6.47	6.47	6.47	6.47
Digestible Methi. + cystine	g/kg	9.08	9.08	9.08	9.08	9.08
Digestible Lysine	g/kg	11.07	11.07	11.07	11.07	11.07
Digestible Threonine	g/kg	6.75	6.75	6.75	6.75	6.75
Digestible Tryptophan	g/kg	2.07	2.07	2.07	2.07	2.07
Digestible Valine	g/kg	7.98	7.98	7.98	7.98	7.98

The treatments mentioned above were subjected to three different temperatures (24, 30 and 36°C).

1 Mineral Premix (concentration/kg of product): Mn - 60 g, Fe - 80 g, Zn - 50 g, Cu - 10 g, Co - 2 g, I - 1 g and Se - 250 mg.

2 Vitamin Premix (concentration/kg of product): Vit. A - 15 mil UI, Vit. D3 - 1,500,000 UI. Vit. E - wm 15000; Vit.B1 - 2.0 g, Vit. B2 - 4.0 g Vit. B6 - 3.0 g, Vit. B12 - 0015 g, nicotinic acid - 25 g, pantothenic acid - 10 g; Vit.K3 - 3.0 g, folic acid - 1.0 g.

3 Inert = Kaolin.

4 Phytase enzyme = 100 grams/ton provides 500 FTUs/kg of feed.

Phytase added to the experimental diets was from *Escherichia coli* (*E. coli*) produced in *Thricoderma reesei* (Quantum Blue, AB Vista, Marlborough, UK), replacing part of the inert ingredient in the diets.

The treatments consisted of five diets composed of corn and soybean meal, which were supplied in each climatic chamber (24, 30 and 36°C). T1: Treatment 1 (0FTU) consisted of a negative control diet, without phytase supplementation and with reduced calcium and phosphorus concentrations. T2: Treatment 2 (500FTU) was a balanced diet with the addition of phytase, which followed the recommendations for the use of the phytase nutritional matrix for calcium and phosphorus. The other treatments (T3 - 1000FTU; T4 - 1500FTU; T5 - 3000FTU) contained a super-dose of phytase. The concentrations of phytase were achieved by adding phytase to replace inert material, considering the concentrations in the negative control diet. The birds had access to water and feed *ad libitum* throughout the experimental period.

### Productive performance evaluation

The parameters evaluated were feed intake (FI - g/bird/day), total egg production (TEP - %), egg weight (EW - g), egg mass (EM - g/bird/day) and eggshell thickness (ET - mm). The performance evaluation period was divided into two 21-day cycles. At the end of each period, the leftover feed from each plot was collected to calculate feed intake.

Feed intake (FI) was determined by dividing the difference between the feed provided during the treatment phase and the leftover feed weighed at the end of the phase by the number of birds in the plot, and then calculated to obtain the average FI per bird in the plot (g/bird/day).

Eggs were collected twice a day (8:00 a.m. and 3:00 p.m.) and recorded on a laying frequency and mortality form for data correction. Percentage of egg production was obtained by collecting the number of eggs produced daily corrected for mortality, so that the ratio of whole eggs produced was expressed as a percentage for each treatment, over the average number of birds in the period (%/bird/day). This corresponded to the production of marketable eggs.

All whole eggs produced in the last three days of each production cycle were weighed individually by using analytical scales (0.001 g) to obtain the average egg weight, which was then multiplied by the total number of eggs produced in the experimental period, thus obtaining the total egg mass. This mass was divided by the total number of birds per day, and expressed in grams of egg/bird/day.

At the end of each production cycle, four eggs were selected per experimental plot. They were broken in half (equatorial region), the shells were washed and sent to be dried in an oven at 55°C for a period of 24 h. The eggshell thickness (ET) was subsequently analyzed and determined with the aid of a digital micrometer (Mitutoyo, resolution 0.001 mm) on the median line of the egg.

### Blood serum biochemistry

For blood biochemistry, the birds were submitted to fasting for 6 h, and then 4 ml of blood was collected from 1 bird per experimental plot by puncturing the jugular vein with a 13 × 0.4 mm needle. The blood was stored in a dry tube with a clot activator (BD Vacuteiner® Seco), and after 30 min it was centrifuged at 3,500 rpm in a centrifuge (SL-702/RAF30, Solab) for 1 min to obtain the serum, which was later stored in 2 ml Ependorf tubes and frozen for posterior analysis.

Serum levels of gamma glutamyl transferase (Bioclin Kit, Reference k080-2), alkaline phosphatase (Bioclin Kit, Reference k224-2), phosphorus (Bioclin Kit, Reference #K020-1), calcium (Bioclin Kit, Reference #K051-2), creatine kinase (CK) (Bioclin Kit, Reference #K010-1.1) and uric acid (Bioclin Kit, Reference k139-2) were analyzed at the Clinical Pathology Laboratory of the Veterinary Hospital of the University of Trás-os-Montes and Alto Douro (UTAD), Vila Real, Portugal. The clinical biochemistry analyzer respons®920 - DiaSys Diagnostic Systems GmbH - Germany, which uses colorimetry and immunoturbidimetric assay, was used for the analyses.

### Organ harvesting

At the end of the 2nd production cycle, 6 birds per treatment were randomly selected and euthanized by electronarcosis for organ collection: small intestine (duodenum, jejunum), kidney, and uterus, for real-time PCR (qPCR) analysis for the calcium transporter Calbindin-D28k. In addition, the collected kidneys were also used for TRPV6 analysis.

### Real-time PCR (qPCR) for Calbindin-D28k

Duodenum, jejunum, kidney, and uterus tissue samples were collected from 6 randomly selected animals in each treatment. Samples were immediately frozen in liquid nitrogen and then transferred to an ultra-freezer at −80°C until processing. Tissues were homogenized in lysis buffer, and RNA extraction was performed by using the SV Total RNA Isolation System kit (Promega). The concentration and purity of RNA samples were determined by absorbance ratios 260/280 and 260/230 obtained by using a spectrophotometer (Colibri Microvolume Spectrometer, Titertek Berthold). Reverse transcription was performed by using GoScript™ Reverse Transcription Mix, Oligo(dT) (Promega) according to the manufacturers' recommendations.

The expression of selected genes was assessed by using a Stratagene Mx3005P qPCR (quantitative polymerase chain reaction) system (Agilent Technologies). Oligonucleotides were obtained from previously published Japanese quail sequences, and HMBS was used as an endogenous control (Table 2). PCR were performed by using Brilliant III Ultra-Fast SYBR® Green qPCR Master Mix low ROX (Agilent Technologies). Amplification conditions were 3 min at 95°C followed by 40 cycles of 15 s at 95°C, and 20 s at 60°C; 1 min at 95°; 30 s at 55°, and finally 30 s at 95°C.

**Table 2:** Sequence of oligonucleotides used for quantitative PCR.

Genes	qPCR primers (5′−3′)	GenBank number	Product size
***CALBINDIN28***	>GACGGCAATGGGTACATGGA <TCGGGTGTTAAGTCCAAGCC	XM_015855985.1	98 bp
***TRPV6***	>CCATCATTGCCACCCTCCTT <AGCAACAATCTGGGCTCTCC	XM_015873874.1	107 bp
***HMBS***	˃TGACCTGGTAGTTCACTCCTT ˂TTGCAAATAGCACCAATGGTAAAG	XM_015872688.2	123 bp
	˃ = forward; ˂ = reverse		

Dissociation curve analysis allowed the evaluation of primer specificity. Samples were analyzed in duplicate, and relative quantification (target gene/endogenous control) determined their expression. Data were normalized to a calibrator sample by using the ΔΔCt method (Pfaffl, 2001) with correction for amplification efficiency.

### Statistical analyses

The data were subjected to an Analysis of Variance (ANOVA) using the statistical software R version 4.2.0 (R Core Team, 2022) to determine the effects of different phytase levels and temperature ranges on the measured variables. For variables showing significant differences (*P* < 0.05), Tukey's test was conducted to compare the means across different temperatures. In addition, regression analysis was employed to identify the optimal phytase level.

The variables were analyzed according to the following mathematical model:

$$Y_{ijkl}=\mu +\alpha_{i}+\beta_{j}+{\left(\alpha \beta \right)}_{ij}+\varepsilon_{ijkl}$$

Where:

• Y_ijkl_ = response variable
• μ = overall mean
• α_i_ = effect of the i th level of phytase
• β_j_ = effect of the j-th temperature range
• (αβ)_ij_ = interaction effect between the i th level of phytase and the j-th temperature range
• ϵ_ijkl_= random error term associated with each observation, assumed to be normally distributed with mean zero and constant variance

in case of significance for phytase the following model was applied:

$$Y_{i}=\beta_{0}+\beta_{1}X_{i}+\varepsilon_{i}$$

Where:

• Y_i_ = the response variable for the i th observation.
• β_0_ = the intercept, representing the expected value of Y when phytase is at zero (baseline level).
• X_i_ = phytase level for the i th observation.
• β_1_ = the slope, indicating the effect of a one-unit increase in phytase on the response variable.
• ϵ_i_= random error term for the i th observation, assumed normally distributed with mean zero and constant variance.

## Results

### Performance

In the results of feed intake (FI - g/bird/day), total egg production (TEP - %), egg weight (EW - g), egg mass (EM - g/bird/day), and eggshell thickness (ET - mm) (Table 3), we can observe a significant effect for feed intake, only among the temperatures analyzed (*p* < 0.0001). In them, the animals submitted to a temperature of 36°C had lower feed intake when compared to the animals submitted to the other temperatures (24 and 30°C).

**Table 3:** Mean values of feed intake (FI), total egg production (TEP), egg weight (EW) Egg mass (EM) and Eggshell thicknesses (ET) of Japanese quails fed with different doses of phytase and subjected to three temperatures.

Parameters	Temperature	Phytase (FTU/Kg)					Mean	CV%	*p-Value*			
		0	500	1000	1500	3000						
									Temperature	Phytase	Temp x Phy	Regression
FI, g/bird/day	24	18.931	19.701	19.596	19.484	19.849	19.512a		<0.0001	0.3491	0.9608	NS
	30	19.705	19.978	20.309	19.997	19.407	19.879a	7.737				
	36	15.024	16.179	16.484	16.118	15.471	15.855b					
	Mean	17.887	18.619	18.796	18.533	18.242						
TEP, %	24	66.013	66.749	68.071	70.074	65.365	67.254b		<0.0001	0.7607	0.9266	NS
	30	80.549	76.297	82.280	80.740	77.122	79.398a	11.670				
	36	66.379	64.943	64.313	61.510	62.570	63.943b					
	Mean	70.980	69.330	71.555	70.775	68.352						
	24	10.560	11.109	11.058	10.875	11.103	10.941					
EW, g	30	10.818	10.967	10.553	10.963	10.999	10.860	17.034	0.3002	0.3903	0.3168	NS
	36	9.267	9.666	12.624	9.957	9.823	10.268					
	Mean	10.215	10.581	11.412	10.599	10.642						
EM, g/bird/day	24	6.955	7.413	7.519	7.619	7.241	7.349b					
	30	8.711	8.369	8.680	8.851	8.470	8.616a	21.494	<0.0001	0.4463	0.7208	NS
	36	6.209	6.288	8.297	6.129	6.170	6.619b					
	Mean	7.292	7.357	8.165	7.533	7.294						
ET, mm	24	0.427a	0.406a	0.410a	0.404b	0.406a	0.411		0.1180	0.0329	<0.0001	NS
	30	0.402b	0.395a	0.411a	0.399b	0.407a	0.403	3.6018				
	36	0.387b	0.398a	0.394a	0.443a	0.412a	0.407					
	Mean	0.405	0.399	0.405	0.415	0.408						

CV% = Coefficient of variation; NS = Not significant. Means followed by different letters in the columns compare temperatures within each phytase concentration, by Tukey's test (*p* < 0.05). Treatment 1 (0FTU); Treatment 2 (500FTU); Treatment 3 (1000FTU); Treatment 4 (1500FTU); Treatment 5 (3000FTU).

For total egg production (*p* < 0.0001) and egg mass (*p* < 0.0001), we also noticed effects only among the temperatures analyzed, highlighting that the animals subjected to temperatures of 30°C obtained greater total production and egg mass, when compared to animals subjected to other temperatures (24 and 36°C).

For the ET variable, we can observe a significant effect in the interaction between phytase levels vs. temperature (*P* < 0.0001) on the shell thickness variable. We can observe that birds not supplemented with Phytase at higher temperatures (30 and 36°C) have lower ET when compared to birds at comfort temperature (24°C). However, when furnished in the diet, phytase provided a better ET mainly at higher temperatures (30 and 36°C), obtaining even a higher ET at a level of 1500 FTU at the temperature of 36°C. No significant effect was observed for the other variables analyzed.

### Serum biochemistry

For the variables of Gamma Glutamyl Transferase (GGT); Alkaline Phosphatase (ALP); Phosphorus (P); Calcium (Ca); Creatine Kinase (CK) and Uric Acid (UA) (Table 4), we can observe an effect for ALP among the analyzed temperatures. When subjected to temperatures of 36°C, birds had higher values for ALP compared to 24°C and 30°C (*p* = 0.01). An increasing linear effect was also observed (Table 6) for the levels of ALP (*p* = 0.0404) as the levels of phytase in the diets increased.

**Table 4:** Gamma Glutamyl Transferase (GGT); Alkaline Phosphatase (ALP); Phosphorus (P); Calcium (Ca); Creatine Kinase (CK); Uric Acid (UA), of Japanese quails fed diets containing phytase super-dosing and subjected to different temperatures.

Parameters	Temperature	Phytase (FTU/Kg)					Mean	CV%	*p-Value*			
		0	500	1000	1500	3000						
									Temperature	Phytase	Temp x Phy	Regression
GGT (U/L)	24	0.37	1.38	3.34	5.22	0.58	2.18	25.89	0.84	0.28	0.60	NS
	30	0.67	4.12	1.10	0.37	0.66	1.38					
	36	0.67	7.30	1.10	0.52	0.41	1.99					
	Mean	0.57	4,27	1,85	2,03	0,55						
ALP (U/L)	24	260.6	303.8	262.7	260.0	303.9	278.2B	27.98	0.01	0.01	0.91	0.0404*
	30	199.1	300.5	212.1	223.8	321.7	251.5B					
	36	284.6	392.6	314.5	354.9	369.8	343.3A					
	Mean	248.1	332.3	263.1	279.6	331.8						
P (mg/dl)	24	5.20	5.05	5.22	5.71	5.29	5.29A	19.38	0.01	0.82	0.83	NS
	30	4.61	4.54	4.38	4.16	4.84	4.50B					
	36	4.92	4.47	3.91	4.22	4.20	4.34B					
	Mean	4.91	4.68	4.50	4.70	4.77						
Ca (mg/dl)	24	19.18	18.12	18.50	21.45	19.88	19.42A	12.00	0.02	0.19	0.17	NS
	30	18.73	16.05	17.62	19.01	16.34	17.55B					
	36	17.28	16.68	16.92	15.65	17.40	16.78B					
	Mean	18.40	16.95	17.68	18.70	17.87						
CK (U/L)	24	770.7	564.4	748.6	539.4	660.5	656.7	26.61	0.94	0.05	0.37	NS
	30	741.6	740.2	748.3	485.1	674.2	677.9					
	36	521.2	683.2	755.7	567.6	799.6	665.5					
	Mean	677.8	662.6	750.9	530.7	711.4	677.8					
UA (mg/dl)	24	3.49A	2.78A	3.99A	3.87A	3.24A	3.47	20.14	0.02	0.15	0.01	NS
	30	3.28A	2.75A	2.41B	3.14A	3.25A	2.97					NS
	36	4.04A	3.60A	3.93A	3.01A	3.23A	3.56					0.0508*
	Mean	3.60	3.04	3.44	3.34	3.24						

CV% = Coefficient of variation; NS = Not significant. Means followed by different letters in the columns compare temperatures within each phytase concentration by Tukey's test (*p* < 0.05). * Linear Effect (ALP; *y* = 0.0185x + 268.75; R^2^: 0.2972); * Linear Effect (UA; *y* = −0.0003x + 3.8874; R^2^: 0.4993); Treatment 1 (0FTU); Treatment 2 (500FTU); Treatment 3 (1000FTU); Treatment 4 (1500FTU); Treatment 5 (3000FTU).

The results obtained for P and Ca demonstrated an effect only for temperatures. Animals subjected to temperatures of 24°C presented higher values ​​for P and Ca compared to birds subjected to temperatures of 30 and 36°C.

Regarding blood UA concentration, we observed a significant impact (*P* = 0.01) in the interaction between temperatures and phytase concentrations. When added to the birds' diets, the phytase enzyme provided similar UA values, except at the temperature of 30°C at a level of 1000 FTU, where the animals presented lower UA values. A decreasing linear effect (Table 6) in UA concentrations (*P* = 0.0508) was also observed in birds kept at temperatures of 36°C as the enzyme levels in the birds' diets increased.

### Calbindin-D28 K and TRPV6 gene expression

**Fig. 1:**
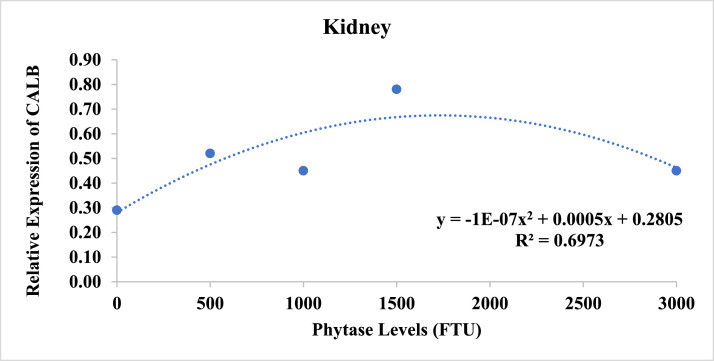
Expression of the Calbindin-D28 K gene in the kidneys of Japanese quails fed different doses of phytase and subjected to three temperatures.

The results of Calbindin-D28 K gene expression (Table 5) revealed a significant quadratic effect (*P* = 0.0202) of phytase concentrations on gene expression in the kidneys (Fig. 1). Increasing the dietary phytase, Calbindin-D28 K expression also increased, reaching its peak at 2500 FTU of phytase (Table 6). No effect was observed for the different temperatures analyzed.

**Table 5:** Calbindin-D28 K gene expression in the duodenum, jejunum, kidneys, and uterus of Japanese quails fed diets containing phytase super-dosing and subjected to different temperatures.

Parameters	Temperature	Phytase (FTU/Kg)					Mean	CV%	*p-Value*			
		0	500	1000	1500	3000						
Expression									Temperature	Phytase	Temp x Phy	Regression
	24	0.53	0.55	0.32	0.56	0.46	0.48		0.4650	0.3016	0.8182	NS
Duodenum	30	0.13	0.28	0.28	0.72	0.60	0.40	15.90				
	36	0.21	0.39	0.31	0.50	0.26	0.33					
	Mean	0.29	0.41	0.31	0.59	0.44						
	24	0.73	1.00	0.83	1.48	0.96	1.00			0.0185		NS
Jejunum	30	0.44	0.74	0.87	1.71	0.58	0.87	21.40	0.1619		0.9935	
	36	0.32	0.54	0.60	1.08	0.37	0.58					
	Mean	0.49	0.76	0.77	1.43	0.64						
	24	0.47	0.74	0.47	0.85	0.40	0.58					
Kidney	30	0.18	0.46	0.44	0.75	0.31	0.43	14.30	0.4069	0.0311	0.8788	0.0202**
	36	0.22	0.36	0.45	0.75	0.64	0.48					
	Mean	0.29	0.52	0.45	0.78	0.45						
	24	0.51	0.36	0.67	0.51	0.20	0.45a		0.0013	0.7033	0.6184	NS
Uterus	30	0.30	0.26	0.21	0.50	0.47	0.35a	14.10				
	36	0.08	0.06	0.06	0.17	0.04	0.08b					
	Mean	0.30	0.23	0.31	0.39	0.24						

CV% = Coefficient of variation; NS = Not significant. Means followed by different letters in the columns compare temperatures within each phytase concentration by Tukey's test (*p* < 0.05). ** Quadratic Effect (*y* = −1E-07 × 2 + 0.0005x + 0.2805; R^2^: 0.6973), Maximum Point 2500 FTU. Treatment 1 (0FTU); Treatment 2 (500FTU); Treatment 3 (1000FTU); Treatment 4 (1500FTU); Treatment 5 (3000FTU).

**Table 6:** Regression equations of the parameters of alkaline phosphatase (ALP); uric acid (UA) and expression of Calbidin-D28 K in the kidney of Japanese quails fed diets containing phytase super-dosing, in different thermal environments.

Parameter	Effect	Equation	R^2^	Level
ALP (U/L)	Linear	*y* = 0,0185x + 268,75	0,2972	-
UA (mg/dl)	Linear	*y* = −0,0003x + 3,8874	0,4993	-
Kidney	Quadrático	*y* = −1E-07×^2^ + 0,0005x + 0,2805	0,6973	2500 FTU ^Max^

**Fig. 2:**
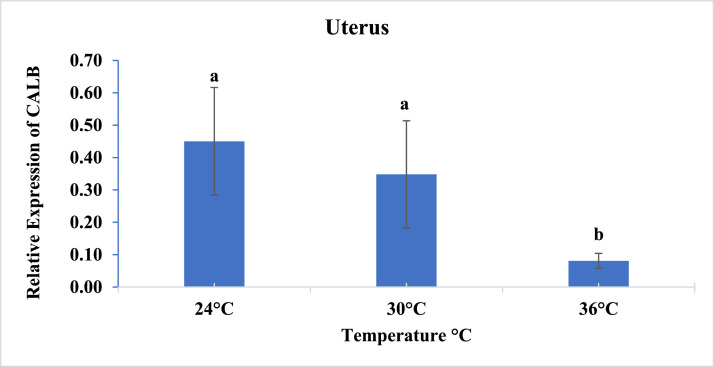
Expression of the Calbindin-D28 K gene in the uterus of Japanese quails fed different doses of phytase and subjected to three temperatures.

The expression of Calbindin-D28 K in the quail uterus was affected only by the different temperatures analyzed (*P* = 0.0013). Birds exposed to severe heat stress (36°C) showed lower expression of the gene compared to those kept at 24°C and 30°C (Fig. 2). In the other tissues analyzed, no significant effect on gene expression was observed.

Regarding the expression of TRPV6 in the kidneys of quails (Table 7), no significant effect was observed for the temperatures evaluated, nor on the interaction or regression.

**Table 7:** TRPV6 gene expression in the kidney of Japanese quails fed diets containing phytase super-dosing and subjected to different temperatures.

Parameters	Temperature	Phytase (FTU/Kg)							*p-Value*			
		0	500	1000	1500	3000	Mean	CV%				
Expression									Temperature	Phytase	Temp x Phy	Regression
	24	0.359	0.718	0.514	0.475	0.534	0.520					
Kidney	30	0.177	0.258	0.481	0.770	0.244	0.386	14.00	0.0947	0.1611	0.5555	NS
	36	0.164	0.253	0.246	0.385	0.372	0.284					
	Mean	0.233	0.410	0.414	0.544	0.384						

CV% = Coefficient of variation; NS = Not significant. Means followed by different letters in the columns compare temperatures within each phytase concentration by Tukey's test (*p* < 0.05). Treatment 1 (0FTU); Treatment 2 (500FTU); Treatment 3 (1000FTU); Treatment 4 (1500FTU); Treatment 5 (3000FTU).

## Discussion

### Performance parameters

Heat stress (HS) has been a growing concern for poultry producers, especially in tropical climate regions, due to its detrimental impacts on laying hens. This type of stress causes a series of physiological changes, such as oxidative stress, acid-base imbalance, and compromised immune system. These changes result in increased mortality rate, decreased feed efficiency, and reduction in body weight, feed intake, egg production, and bone development (Ribeiro et al., 2024).

In our research, exposing birds to high temperatures proved to be highly detrimental to production. Quails subjected to severe heat stress (36°C) showed a significant reduction in feed intake, resulting in insufficient intake of nutrients and vitamins, which are essential for bird development, especially with regard to bone formation. The effect of temperature on feed intake (FI) was similar to that found by Santos et al. (2019), who reported a reduction in the intake of Japanese quails exposed to high temperatures (35°C). Similarly, Li et al. (2015) and Kapetanov et al. (2015) reported that during heat stress, hens reduced feeding time to decrease heat production, which may indicate reduced FI. When evaluating the effect of heat stress on laying quails (*Coturnix coturnix japonica*), Akdemir et al. (2019), stated that birds exposed to 34°C had lower feed intake, lower egg production and weight, in addition to worse feed conversion.

For laying quails, the thermal comfort range is between 21°C and 27°C (Alagawany et al., 2017). In our research, we observed that animals exposed to 30°C presented greater total production and egg mass compared to those kept at 24°C and 36°C. This finding is interesting because, although 30°C is outside the comfort range, it seems to provide a condition in which quails can optimize their production, possibly due to their greater tolerance to heat. Unlike laying hens and broilers, laying quails have a more favorable surface area:body volume ratio, which increases their ability to dissipate endogenous heat (Porto et al., 2021). Even though 30°C is above ideal, they can tolerate this temperature, although they need a greater intake of vitamins and minerals to compensate for the metabolic changes caused by heat stress (Truong et al., 2023).

Several nutritional strategies have been used to mitigate the negative effects of HS on bone health in birds (Hosseini-Vashan et al., 2016; Yan et al., 2019; Zhang et al., 2021; Ribeiro et al., 2024). Phytase stands out as a proposal to mitigate the effects of heat stress, because in addition to enabling the degradation of the phytate molecule present in the ingredients of the diets, releasing phosphorus and calcium (Hirvonen et al., 2019), important for the body and bone development of animals (Lee et al., 2019), it also releases minerals and vitamins in the process, such as zinc and vitamin D, which are linked to bone development (Zhang et al., 2020, 2021) and also act as antioxidant factors, removing free radicals and protecting cell membranes from oxidative stress caused by high temperatures (Ribeiro et al., 2024).

The use of increasing concentrations of phytase in our study had a positive impact on eggshell thickness, a result directly associated with the increased availability and retention of essential minerals, such as calcium and phosphorus, provided by the action of phytase in the breakdown of phytate. In addition, dietary supplementation with 1500 FTU of phytase not only minimized the deleterious effects of heat stress (36°C), but also improved egg quality by increasing eggshell thickness.

### Blood biochemistry

The evaluation of serum biochemical parameters is essential to understand the physiological responses of quails under different environmental and dietary conditions. Gamma-glutamyl transferase (GGT), a membrane enzyme mainly associated with the biliary and renal epithelia in birds, is a relevant marker for monitoring liver function. In our research, serum GGT levels remained within the normal range for birds (0-10 U/L) (Scholtz et al., 2009; Rezende, 2017), suggesting the absence of active liver injury in quails, even under heat stress conditions.

Another important marker is alkaline phosphatase (ALP), a key enzyme in calcium and phosphorus metabolism in birds. It plays an important role in chondrogenic (cartilage formation) and osteoblastic (bone formation) activities, essential for the proper growth of these species. ALP has low activity in the liver of birds, and its increase in serum or blood plasma is strongly associated with osteoblastic activity and various bone changes, such as accelerated growth, fracture consolidation, osteomyelitis, secondary nutritional hyperparathyroidism, and neoplasms (Barbosa et al., 2011).

Under heat stress, bone metabolism in Japanese quails can be significantly affected, resulting in elevations in alkaline phosphatase (ALP) levels. This increase can be attributed to two main factors: the intensification of osteoblastic activity, which is responsible for the formation of new bone tissue, and the release of ALP by bone cells in response to stress. Elevated osteoblastic activity may occur as an attempt by the body to compensate for the mobilization of calcium and phosphorus, which are essential for the formation of the eggshell, and may be compromised in conditions of extreme heat (Franzini et al., 2022).

The inclusion of phytase in diets can significantly improve the bioavailability of phosphorus and calcium, since the enzyme breaks down the phytic acid present in plant ingredients, releasing inorganic phosphorus and calcium that can be absorbed and used by the body. This increase in the availability of calcium and phosphorus stimulates bone metabolism, promoting adequate bone mineralization and, consequently, increasing the activity of enzymes related to this process, such as alkaline phosphatase. The increasing linear effect on ALP levels with increasing phytase levels observed in our study can be attributed to the greater release of calcium and phosphorus by the action of the phytase enzyme, which improves the availability of these essential minerals for bone metabolism, favoring the development and maintenance of skeletal health in birds.

For laying quails, calcium (Ca) and phosphorus (P) are essential, and their availability is most crucial during the laying period. Calcium and phosphorus, among all minerals, play a fundamental role in the construction of the skeleton, constituting 80 to 85 % of its structure. They are essential in the formation of eggshells and muscle development. Thus, these minerals are indispensable for the proper functioning of the animal's body (Ribeiro et al., 2024).

In our study, serum Ca and P levels of birds kept at temperatures of 30 and 36°C were slightly below the recommendations (Ca: 20 to 30 mg/dL and P: 5 to 7 g/L) (Thrall et al., 2004). This may be explained by the fact that in high temperature environments (36°C), birds tend to reduce feed intake, thus limiting the availability of calcium and phosphorus in the diet. This decrease in intake may affect variations in the absorption and mobilization of these minerals in the blood, since calcium and phosphorus are essential for several physiological functions, including eggshell formation and maintenance of bone health.

When evaluating the effects of acute and chronic heat stress on performance, egg quality, body temperature and blood gas parameters of laying hens, Barrett et al. (2019), observed that, under heat stress, birds tend to have reduced serum Ca levels. Similar results were found in studies with laying hens subjected to heat stress, which demonstrated a decrease in plasma P concentrations in animals exposed to temperatures of 34°C, indicating the occurrence of severe heat stress (Wolfenson et al., 1987; Ait-Boulahsen et al., 1993; Usayran et al., 2001; Persia et al., 2003).

On the other hand, although quails are able to withstand temperatures of up to 30°C, these birds require greater amounts of vitamins and minerals in their diet due to changes in their metabolism (Ribeiro et al., 2024). In this sense, the lower serum levels of Ca and P observed in animals subjected to a temperature of 30°C indicate a greater use of nutrients by the birds to maintain their normal metabolism, since the performance of the birds at this temperature was not inferior to that observed at 24°C.

When evaluating the effect of three temperature ranges (24, 30, and 36°C) on Japanese quails, Ribeiro et al. (2024), confirmed that, at 30°C, the birds showed greater efficiency in the use of dietary calcium compared to those exposed to 24°C, due to a greater demand for this mineral. These findings are in agreement with the results found in our study, demonstrating that quails have a remarkable capacity to adapt to thermal variations, adjusting their metabolism to optimize the use of essential nutrients, such as calcium and phosphorus.

This adaptive capacity is also reflected in the general health status of the birds, including renal function. Uric acid concentrations, which are indicative of renal functionality, were within normal levels in our study (<15 mg/dL) (Thrall et al., 2004; Farias et al., 2021), suggesting that despite the heat stress conditions, the quails were able to maintain adequate renal function. Since uric acid is one of the main indicators of renal tubule integrity, maintaining normal levels reinforces the resilience of birds in situations of thermal variation, without compromising renal health. Furthermore, the results of our study showed that dietary supplementation with phytase not only minimized the negative effects of heat stress (36°C), but, at a level of 1500 FTU at the same temperature, also reduced serum uric acid levels in birds, promoting better renal health even under heat stress conditions.

### Calbindin-D28 K gene expression

Calbindin has been described in studies in its two main forms: calbindin-D9 K, a low molecular weight protein present in the intestine of mammals, and calbindin-D28 K, a high molecular weight protein found in the kidney of mammals (Fleet and Schoch, 2010), as well as in the brain, kidney, intestine, and uterus of birds (Bar, 2009; Fleet and Schoch, 2010) where it facilitates the transport of calcium during the formation of the eggshell (Moraes et al., 2021).

Specifically, calbindin-D28k is an intracellular ionic Ca-binding protein (Ca^2+^), that acts as a Ca transporter from the apical membrane to the basolateral membrane and is involved in Ca^2+^ mobilization in the intestine (absorption), kidney (absorption), and uterine glands (release) (Wang et al., 2022). It demonstrates the ability not only to influence intestinal absorption, but also to modulate the deposition of this mineral in the uterus. This ability potentially affects not only the production, but also the quality of eggshells (Ribeiro et al., 2024).

In the present study, Calbindin-D28 K gene expression was observed in the intestine in all treatments analyzed, corroborating previous findings on its presence in laying hens (Wasserman e Taylor, 1966; Moraes et al., 2021). However, no changes in Calbindin-D28 K gene expression were detected in response to increased temperature or increased phytase levels in the diets. These results suggest that, despite exposure to heat stress, there was no significant change in Calbindin-mediated calcium transport in the intestine. In agreement with our results, Moraes et al. (2021), when investigating the effect of four temperature ranges (20, 24, 28, and 32°C) in laying quails, also found that increased temperature did not influence Calbindin-D28 K gene expression in the intestine of these birds.

The kidney is a crucial organ in the dynamic regulation of mineral homeostasis during the oviposition cycle. It plays a central role in the reabsorption and excretion of calcium and phosphorus. It guarantees the balance of these minerals in the blood, ensuring that adequate levels are maintained to support physiological demands such as eggshell formation and maintenance of bone health (Sinclair-Black et al., 2024). The results of this study indicate that in the kidney, Calbindin-D28 K gene expression was positively modulated by dietary phytase levels. The inclusion of phytase is essential for the breakdown of the phytate molecule (the main storage form of phosphorus in plant ingredients), releasing not only phosphorus but also increasing the availability of other important nutrients, such as calcium, other minerals, and vitamins. Among these vitamins, D3 in its metabolically active form, 1,25(OH)₂D₃, is crucial for the absorption and utilization of calcium by birds (Moraes et al., 2021; Ribeiro et al., 2024).

In the kidneys, when 1,25(OH)₂D₃ binds to VDR (Vitamin D Receptor), it activates this molecule, allowing it to move from the cytoplasm to the cell nucleus, where it binds to specific regions of DNA (Chacar et al., 2020; Wu et al., 2022). This interaction stimulates the expression of genes responsible for calcium absorption, such as Calbindin-D28 K, which plays a fundamental role in the transport and regulation of calcium in kidney cells, facilitating the reabsorption of this mineral, which is essential for maintaining bone health and egg production (Moraes et al., 2021). In our study, increasing levels of phytase supplementation was effective in elevating the expression of this transporter. 1500 FTU provided the highest level of Calbindin-D28 K expression in quail kidneys.

The uterus is an essential organ in the formation of the eggshell, acting in the mobilization and deposition of calcium, mainly in the form of calcium carbonate (CaCO₃), for the mineralization of the shell (Andrade et al., 2023). This process is essential to ensure the rigidity and integrity of the shell, providing adequate protection to the eggs. For uterine tissue, the gene expression of Calbindin-D28k was affected by temperatures. At high temperatures (36°C), there is a reduction of the presence of the calcium transporter Calbindin-D28 K in the uterus, due to the lower conversion of vitamin D3 into its metabolically active form, 1,25(OH)₂D₃ (Moraes et al., 2021). This metabolite is essential for the absorption and utilization of calcium, and heat stress compromises this process, resulting in lower expression of Calbindin-D28 K in the uterus, which affects calcium deposition in eggshell formation (Ebeid et al., 2012). Our findings confirm that at elevated temperatures, Calbindin-D28 K expression is reduced, which impairs calcium deposition in eggshell formation and impacts its quality.

### TRPV6 gene expression

The TRPV6 ion channel (Transient Receptor Potential Vanilloid Channel Type 6) acts as an epithelial calcium channel in organs and glands characterized by high demand for calcium transport. According to some studies (Brown et al., 2005; Bianco et al., 2007), this ion channel has a facilitating effect on the entry of calcium into epithelial cells, expressed in the intestinal and renal absorption and reabsorption epithelia, but there is still little information about its expression pattern in laying quails (Moraes et al., 2021). In the present study, TRPV6 gene expression was observed in the kidney in all treatments analyzed, corroborating previous findings on its presence in laying quails (Moraes et al., 2021). However, no changes in TRPV6 gene expression were detected in response to increased temperature or to the effect of phytase doses used. Thus, even with phytase supplementation or temperature variations, TRPV6 may not require a greater expression to maintain efficient calcium absorption.

## Conclusion

Phytase supplementation brought significant benefits to birds, especially those kept at temperatures of 30°C, due to the increased demand for vitamins and minerals caused by metabolic changes. Phytase improved the efficiency of calcium (Ca) absorption by stimulating increased expression of calbindin-D28 K in the kidneys, which favored increased Ca reabsorption to be used in total egg production. In addition, dietary supplementation with 1500 FTU of phytase not only minimized the deleterious effects of heat stress (36°C), but also increased eggshell thickness, improving egg quality, reducing serum uric acid levels, and reflecting on better kidney health.

## Disclosures

The authors declare that they have no known competing financial or personal interest that could have impact the outcome of the study reported in this paper.

## References

[bib0001] Ait-Boulahsen A., Garlich J.D., Edens F.W. (1993). Calcium deficiency and food deprivation improve the response of chickens to acute heat stress. J. Nutr..

[bib0002] Akdemir F., Köseman A., Şeker I. (2019). Alchemilla vulgaris effects on egg production and quality expressed by heat-stressed quail during the late laying period. S. Afr. J. Anim. Sci..

[bib0003] Alagawany M., Farag M.R., ABD El-Hack M.E., Patra A. (2017). Heat stress: effects on productive and reproductive performance of quail. World. Poult. Sci. J..

[bib0004] Andrade K.G., Cruz F.K., Kaneko I.N., Nascimento M.C., Iwaki L.C. (2023). Santos. Daily egg-cycle in Japanese quail: serum biochemistry, bones, and oviduct changes. Braz. J. Poult. Sci..

[bib0005] Babatunde O.O., Cowieson A.J., Wilson J.W., Adeola O. (2019). The impact of age and feeding length on phytase efficacy during the starter phase of broiler chickens. Poult. Sci..

[bib0006] Bar A. (2009). Calcium transport in strongly calcifying laying birds: mechanisms and regulation. Comp. Biochem. Physiol. A Mol. Integr. Physiol..

[bib0007] Barbosa T.S., Mori C.K., Polônio L.B., Ponsano E.H.G., Ciarlini P.C. (2011). Serum biochemical profile of laying hens in the region of Araçatuba. SP. Semina: Ciênc. Agrár..

[bib0008] Barrett N.W., Rowland K., Schmidt C.J., Lamont S.J., Rothschild M.F., Ashwell C.M., Persia M.E. (2019). Effects of acute and chronic heat stress on the performance, egg quality, body temperature, and blood gas parameters of laying hens. Poult. Sci..

[bib0009] Beggs M.R., Alexander R.T. (2017). Intestinal absorption and renal reabsorption of calcium throughout postnatal development. Exp. Biol. Med. (Maywood).

[bib0010] Bernardes R.D., Oliveira C.H., Calderano A.A., Ferreira R.S., Dias K.M.M., Almeida B.F., Aleixo P.E., Albino L.F.T. (2022). Effect of phytase and protease combination on performance, metabolizable energy, and amino acid digestibility of broilers fed nutrient-restricted diets. Rev. Bras. Zootec..

[bib0011] Bianco S.D., Peng J.B., Takanaga H., Suzuki Y., Crescenzi A., Kos C.H., Zhuang L., Freeman M.R., Gouveia C.H.A., Wu J., Luo H., Mauro T., Brown E.M., Hediger M.A. (2007). Marked disturbance of 99calcium homeostasis in mice with targeted disruption of the Trpv6 calcium channel gene. J. Bone Miner. Res..

[bib0012] Brown A.J., Krits I., Armbrecht H.J. (2005). Effect of age, vitamin D, and calcium on the regulation of rat intestinal epithelial calcium channels. Arch. Biochem. Biophys..

[bib0013] Chacar F.C., Kogika M.M., Zafalon R.V.A., Brunetto M.A. (2020). Vitamin D metabolism and its role in mineral and bone disorders in chronic kidney disease in humans, dogs, and cats. Metabolites.

[bib0014] Dallmann H.M., Avila V.S., Krabbe E.L., Surek D., Bedendo G.C., Toledo T.S., Dallmann P.R., Roll A.A.P., Roll V.F.B., Rutz F. (2023). Different phytase levels and energy densities in broiler diets on performance, nutrient digestibility, and bone integrity from 28 to 35 days of age. Arq. Bras. Med. Vet. Zootec..

[bib0015] Ebeid T.A., Suzuki T., Sugiyama T. (2012). High ambient temperature influences eggshell quality and calbindin-D28k localization of eggshell gland and all intestinal segments of laying hens. Poult. Sci..

[bib0016] Farias M.R.S., Leite S.C.B., Silva H.P., Pacheco D.B., Alves G.C., Abreu C.G., Freitas E.R. (2021). Superdosing phytases in the diets of light laying hens: productive performance and bone quality. Braz. J. Poult. Sci..

[bib0017] Fleet J.C., Schoch R.D. (2010). Molecular mechanisms for regulation of intestinal calcium absorption by vitamin D and other factors. Crit. Rev. Clin. Lab. Sci..

[bib0018] Franzini B.D., Cruz L.C.F., Sampaio S.A., Borges K.F., Barros H.S.S., Santana F.X.C., Gouveia A.B.V.S., Paulo L.M., Minafra C.S. (2022). Blood hematological and hormonal indicators of stress in poultry. Res. Soc. Dev..

[bib0019] Hirvonen J., Liljavirta J., Saarinen M.T., Lehtinen M.J., Ahonen I., Nurminen P.I. (2019). Effect of phytase on in vitro hydrolysis of phytate and the formation of myo-inositol phosphate esters in various feed materials. J. Agric. Food Chem..

[bib0020] Hosseini-Vashan S.J., Golian A., Yaghobfar A. (2016). Growth, immune, antioxidant, and bone responses of heat stress-exposed broilers fed diets supplemented with tomato pomace. Int. J. Biometeorol..

[bib0021] Kapetanov M., Pajic M., Ljubojevic D., Pelic M. (2015). Heat stress in the poultry industry. Arch. Vet. Med..

[bib0022] Lee S.A., Nagalakshmi D., Raju M.V.L.N., Rao S.V.R., Bedford M.R., Walk C.L. (2019). Phytase as an alleviator of high-temperature stress in broilers fed adequate and low dietary calcium. Poult. Sci..

[bib0023] Li M., Wu J., Chen Z. (2015). Effects of heat stress on the daily behavior of Wenchang chickens. Braz. J. Poult. Sci..

[bib0024] Manobhavan M., Elangovan A.V., Sridhar M., Shet D., Ajith S., Pal D.T., Gowda N.K.S. (2016). Effect of super dosing of phytase on growth performance, ileal digestibility, and bone characteristics in broilers fed corn–soya-based diets. J. Anim. Physiol. Anim. Nutr..

[bib0025] Martínez-Vallespín B., Männer K., Ader P., Zentek J. (2022). Evaluation of high doses of phytase in a low-phosphorus diet in comparison to a phytate-free diet on performance, apparent ileal digestibility of nutrients, bone mineralization, intestinal morphology, and immune traits in 21-day-old broiler chickens. Anim. (Basel).

[bib0026] Moraes L.R., Delicato M.E.A., Cruz A.S., Silva H.T.F.N.P., Alves C.V.B.V., Campos D.B., Saraiva E.P., Costa F.G.P., Guerra R.R. (2021). Methionine supplementing effects on intestine, liver, and uterus morphology, and on positivity and expression of Calbindin-D28k and TRPV6 epithelial calcium carriers in laying quail in thermoneutral conditions and under thermal stress. PLoS ONE.

[bib0027] Persia M.E., Utterback P.L., Biggs P.E., Koelkebeck K.W., Parsons C.M. (2003). Interrelationship between environmental temperature and dietary nonphytate phosphorus in laying hens. Poult. Sci..

[bib0028] Pfaffl M.W. (2001). A new mathematical model for relative quantification in real-time RT–PCR. Nucl. Acid. Res.

[bib0029] Pieniazek J., Smith K.A., Williams M.P., Manangi M.K., Vazquezanon M., Solbak A., Miller M., Lee J.T. (2017). Evaluation of increasing levels of a microbial phytase in phosphorus deficient broiler diets via live broiler performance, tibia bone ash, apparent metabolizable energy, and amino acid digestibility. Poult. Sci..

[bib0030] Pirgozliev V., Bedford M.R., Acamovic T., Mares P., Allymehr M. (2011). The effects of supplementary bacterial phytase on dietary energy and total tract amino acid digestibility when fed to young chickens. Brit. Poult. Sci..

[bib0031] Porto M.L., Teófilo T.S., Cavalcanti D.M.L.P., Freitas C.I.A., Oliveira M.F., Fontenele-Neto J.D. (2021). Incubation variables, performance, and morphometry of the duodenal mucosa of Japanese quails (Coturnix coturnix japonica) submitted to different incubation temperatures and thermally challenged after hatching. Arqu. Bras. Med. Vet. Zootec..

[bib0032] R Core Team (2022). https://www.R-project.org/.

[bib0033] Rezende M.S. (2017). https://repositorio.ufu.br/bitstream/123456789/21136/1/PerfilBioqu%C3%ADmicoSangu%C3%ADneo.pdf.

[bib0034] Ribeiro A.G., Silva R.S., Costa F.S., Silva E.G., Santos Ribeiro J.E., Saraiva E.P., Costa F.G.P., Guerra R.R. (2024). Phytase super-dosing modulates bone parameters and the concentration of the calcium epithelial carrier calbindin-D28k in quails (Coturnix coturnix japonica) under thermal stress. Anim. Prod. Sci..

[bib0035] Rojas I.Y.M., González E.A., Menocal J.A., Santos T.T., Arguello J.R., Coello C.L. (2017). Assessment of a phytase included with lactic acid on productive parameters and on deposition of phosphorus, calcium, and zinc in laying hens fed with sorghum–soybean-meal-based diets. J. Appl. Anim. Res..

[bib0036] Rostagno M.H. (2020). Effects of heat stress on the gut health of poultry. J. Anim. Sci..

[bib0037] Rostagno H.S., Albino L.F.T., Hannas M.I., Donzele J.L., Sakomura N.K., Perazzo F.G., Saraiva A., Teixeira M.L., Rodrigues P.B., de Oliveira R.F., de Toledo Barreto S.L., Brito C.O. (2017). https://edisciplinas.usp.br/pluginfile.php/4532766/mod_resource/content/1/Rostagno%20et%20al%202017.pdf.

[bib0038] Santos T.C., Gates R.S., Tinôco I.F.F., Zolnier S., Rocha K.S.O., Freitas L.C.S.R. (2019). Productive performance and surface temperatures of Japanese quail exposed to different environmental conditions at the start of lay. Poult. Sci..

[bib0039] Scholtz N., Halle I., Flachowsky G., Sauerwein H. (2009). Serum chemistry reference values in adult Japanese quail (Coturnix coturnix japonica) including sex-related differences. Poult. Sci..

[bib0040] Sena T.L., Leite S.C.B., Vasconcelos A.M., Bezerra M.M.R., Abreu C.G., Farias M.R.S., Silveira R.M.F. (2020). Does dietary supplementation with phytases affect the thermoregulatory and behavioral responses of pullets in a tropical environment?. J. Therm. Biol..

[bib0041] Sharma N.K., Choct M., Wu S.B., Smillie R., Morgan N., Omar A.S., Sharma N., Swick R.A. (2016). Performance, litter quality and gaseous odour emissions of broilers fed phytase supplemented diets. Anim. Nutr..

[bib0042] Silva V.C., Nascimento R.S., Lopes Neto J.P., Riberio Miranda J., de Melo Lopes F.F., Furtado D.A. (2022). Bioclimatic zoning for quails in the dry period in the state of Paraíba. Brazil. Rev. Ceres.

[bib0043] Sinclair-Black M., Garcia-Mejia R.A., Blair L.R., Angel R., Arbe X., Cavero D., Ellestad L.E. (2024). Circadian regulation of calcium and phosphorus homeostasis during the oviposition cycle in laying hens. Poult. Sci..

[bib0044] Thrall M.A., Baker D.C., Campbell T.W., DeNicola D.B., Fettman M.J., Lassen E.D., Rebar A., Weiser G. (2004).

[bib0045] Truong L., Miller M.R., Sainz R.D., King A.J. (2023). Changes in Japanese quail (Coturnix coturnix japonica) blood gases and electrolytes in response to multigenerational heat stress. PLoS Clim.

[bib0046] Usayran N., Farran M.T., Awadallah H.H., Al-Hawi I.R., Asmar R.J., Ashkarian V.M. (2001). Effects of added dietary fat and phosphorus on the performance and egg quality of laying hens subjected to a constant high environmental temperature. Poult. Sci..

[bib0047] Wang X., Li P., Zhao J., Jiao H., Lin H. (2022). The temporal gene expression profiles of calcium and phosphorus transporters in Hy-Line Brown layers. Poult. Sci..

[bib0048] Wasserman R.H., Taylor A.N. (1966). Vitamin D3-induced calcium-binding protein in chick intestinal mucosa. Science.

[bib0049] Wolfenson D., Sklan D., Graber Y., Kedar O., Bengal I., Hurwitz S. (1987). Absorption of protein, fatty acids and minerals in young turkeys under heat and cold stress. Brit. Poult. Sci..

[bib0050] Wu L., Wang X., Lv X., He L., Qu H., Shi C., Zhang L., Zhang J., Wang Z., Han J. (2022). 1,25-Dihydroxycholecalciferol improved the growth performance and upregulated the calcium transporter gene expression levels in the small intestine of broiler chickens. J. Poult. Sci..

[bib0051] Yan F.F., Mohammed A.A., Murugesan G.R., Cheng H.W. (2019). Effects of a dietary synbiotic inclusion on bone health in broilers subjected to cyclic heat stress episodes. Poult. Sci..

[bib0052] Zhang H., Majdeddin M., Gaublomme D., Taminiau B., Boone M., Elewaut D., Daube G., Josipovic I., Zhang K., Michiels J. (2021). 25-hydroxycholecalciferol reverses heat-induced alterations in bone quality in finisher broilers associated with effects on intestinal integrity and inflammation. J. Anim. Sci. Biotechnol..

[bib0053] Zhang Y.N., Wang S., Li K.C., Ruan D., Chen W., Xia W.G., Wang S.L., Abouelezz K.F.M., Zheng C.T. (2020). Estimation of dietary zinc requirement for laying duck breeders: effects on productive and reproductive performance, egg quality, tibial characteristics, plasma biochemical and antioxidant indices, and zinc deposition. Poult. Sci..

